# Successful medical management of emphysematous gastritis with concomitant portal venous air: a case report

**DOI:** 10.1186/1752-1947-4-140

**Published:** 2010-05-19

**Authors:** Manju Paul, Savio John, Madhav C Menon, Nazar H Golewale, Stan L Weiss, Uma K Murthy

**Affiliations:** 1 Department of Medicine, State University of New York Upstate Medical University, 750 E Adams Street, Syracuse, NY 13202, USA; 2 Department of Radiology, State University of New York Upstate Medical University, 750 E Adams Street, Syracuse, NY 13210, USA

## Abstract

**Introduction:**

The causes of diffuse abdominal pain following pelvic surgery are numerous. We present a rare case of acute abdominal pain in a woman in the post-partum period.

**Case presentation:**

A 25-year-old Caucasian woman with neurofibromatosis type 1 presented to our hospital with diffuse abdominal pain immediately after a cesarean section. The patient was acutely ill and toxic with a fever of 38.8°C, a pulse of 120 beats per minute and a distended abdomen with absent bowel sounds. A computed tomography scan showed air in the wall of the stomach and portal venous system. The patient was successfully treated with intravenous antibiotics, bowel rest and total parenteral nutrition.

**Conclusion:**

It is rare for a case of emphysematous gastritis associated with portal venous air to be treated successfully without surgery. To the best of our knowledge, to date there has been no reported association of emphysematous gastritis with neurofibromatosis.

## Introduction

The causes of diffuse abdominal pain following pelvic surgery are numerous. A strong consideration of a serious intra-abdominal pathology needs to be entertained when imaging studies demonstrate air in the wall of the gastrointestinal organs in patients with fever, distended abdomen and absent bowel sounds in the immediate post-operative period. The importance of early identification of the underlying disease process is illustrated in this rare case of acute abdominal pain in a young female in the post-partum period.

## Case presentation

A 25-year-old Caucasian female with a history of type 1 neurofibromatosis was brought to our hospital with diffuse abdominal pain, nausea, vomiting and fever following cesarean section for fetal distress. She was transferred to our institution within 18 hours of the onset of symptoms for surgical intervention in view of the ominous findings on computed tomography (CT) scan and endoscopy done at the peripheral hospital. She had not passed flatus or stool since surgery and denied hemetemesis, melena, shortness of breath, or chest pain. There was no history of tobacco or alcohol abuse, ingestion of corrosive substances or non-steroidal anti inflammatory drugs (NSAIDs).

Our patient appeared acutely ill and toxic. She had a temperature of 38.8°C, pulse of 120/min, blood pressure of 154/90 mmHg, respiratory rate of 24/min, and oxygen saturation of 97% on 2 L of oxygen. The cardiac and respiratory exams were otherwise unremarkable. Her abdomen was markedly distended. There was diffuse tenderness on palpation of the abdomen with no peritoneal signs. The cesarean section incision appeared clean with no tenderness or discharge. Bowel sounds were absent on auscultation. There were multiple neurofibromas on our patient's neck and anterior chest consistent with her diagnosis of neurofibromatosis. Her white blood cell count was 25,000/mm^3^, with 91% neutrophils. The initial electrolytes, amylase, lipase and liver function tests were within normal limits.

**Figure 1:**
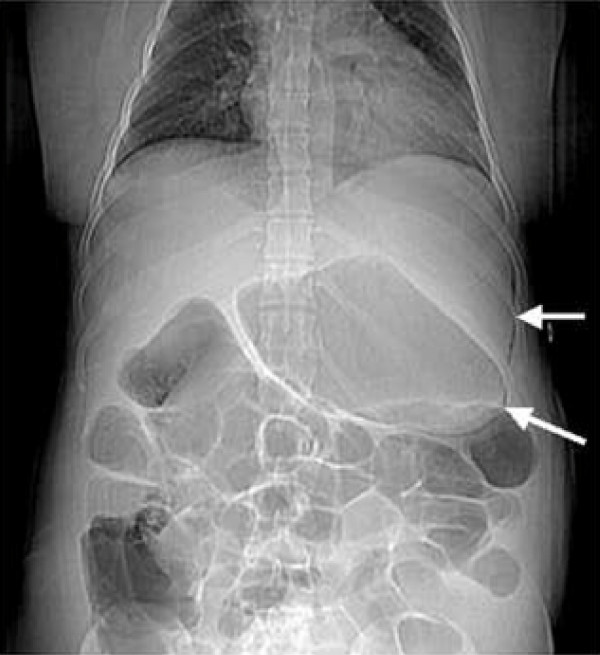
**Scout film showing air along the greater curvature of the stomach**.

**Figure 2:**
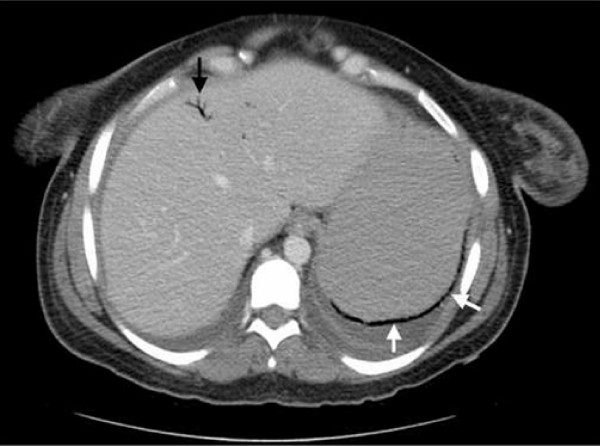
**CT of the abdomen showing air in the stomach wall and portal venous system**. Black arrow: Portal venous air. White arrows: Air in the stomach wall.

CT scan of our patient's abdomen showed marked gastric dilation and air in the wall of the stomach along the entire greater curvature and portal venous system (Figures 1 and 2). There was marked dilatation of the small and large bowel. Esophagogastroduodenoscopy (EGD) of our patient showed areas of diffuse mucosal congestion and extreme pallor as well as ulceration on the posterior wall and greater curvature of the stomach. Gastric biopsy revealed transmural necrosis. *Streptococcus viridans *was isolated from gastric biopsy. Blood cultures did not grow any pathogenic bacteria and nasogastric cultures were not obtained.

**Figure 3:**
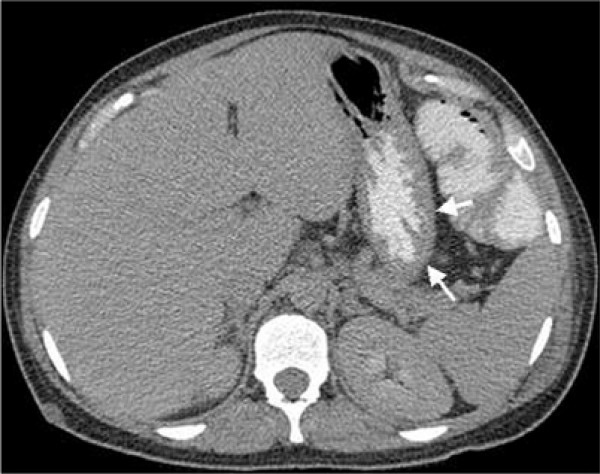
**Follow-up abdominal CT on day eight showing resolution of stomach wall and portal venous air**.

Our patient was diagnosed with emphysematous gastritis and promptly started on intravenous clindamycin and piperacillin/tazobactam, nasogastric decompression and intravenous hydration. Total parenteral nutrition was initiated from day two and our patient was closely monitored in the intensive care unit for three days. She improved with the above measures and tube feedings were initiated from day seven. Follow-up CT scan on day eight showed resolution of the gastric and portal venous air (Figure 3). Our patient was finally discharged home on oral proton pump inhibitors on day 10. A follow-up EGD two months later showed no sequelae and our patient remained asymptomatic.

## Discussion

Differential diagnoses for gas in the wall of the stomach are emphysematous gastritis and gastric emphysema or gastric pneumatosis. Theories suggested for gastric wall air include mechanical, pulmonary, ischemic and bacterial sources [1].

The mechanical theory suggests that gas is forced into the bowel wall through a mucosal defect such as with air insufflation during endoscopy. Our patient had gastric pneumatosis evident on the CT scan even before endoscopy, thus ruling out air insufflation at endoscopy as the source of pneumatosis. The rupture of emphysematous bullae in some patients can cause alveolar air to enter the mediastinum, dissect along the great vessels to the retroperitoneum and through the mesenteric perivascular spaces to reach the bowel wall. Important clues to this clinical situation are the concomitant presence of pneumomediastinum and advanced chronic obstructive pulmonary disease (COPD), which were absent in our patient, thus making a pulmonary process unlikely. Slow-healing mucosal ulcerations caused by ischemia, peptic ulcer disease or inflammatory bowel disease, may also lead to dissection of the luminal gas into the bowel wall [2]. Our patient did not have any episode of peri-operative hypotension and the overall clinical picture did not support an underlying ischemic process.

Emphysematous gastritis is a rare but grave variant of phlegmonous gastritis. It is generally caused by local infection through a mucosal defect by gas-forming microorganisms or via hematogenous dissemination from a distant focus. The stomach is a very uncommon site of involvement, due to its abundant blood supply, acidic pH and efficient mucosal barrier [3]. Most frequently isolated organisms are streptococci, *Escherichia coli*, Enterobacter species, *Pseudomonas aeruginosa *and *Clostridium perfringens *[1]. It has been associated with alcohol abuse, ingestion of corrosive substances, gastroenteritis, diabetes, NSAIDs [4], abdominal surgery, gastric infarction, phytobezoar [5], adenocarcinoma of the stomach [6], leukemia, pancreatitis, disseminated strongyloidiasis in a patient receiving chemotherapy for lymphoma [7], all of which can breach the integrity of the mucosa. Our patient had none of these conditions except the history of recent pelvic surgery. It is possible that a sub-clinical uterine or pelvic sepsis resulting from surgery could have resulted in a hematogenous or transperitoneal infection of the stomach.

Patients with emphysematous gastritis usually present with severe abdominal pain, nausea, vomiting, hemetemesis, low grade fevers and tachycardia [8] as our patient did. Patients with gastric emphysema or gastric pneumatosis generally do not present with acute abdomen, and the prognosis is excellent [1]. Currently, CT scan is the most accurate diagnostic exam [9], although a plain abdominal X-ray can be used as the initial imaging study [10].

It is important to differentiate emphysematous gastritis from gastric emphysema. Early institution of antibiotic therapy covering anaerobes and gram negative bacilli, intravenous hydration and appropriate nutrition is the mainstay of treatment. Emphysematous gastritis usually has a fulminant course with a mortality rate of 60% and gastric strictures are as common as 25% [9]. Surgery should be avoided during the acute phase in the absence of bowel perforation due to friability of the mucosa and the delay in healing of the sutured margins [1]. Air in the portal vein or its radicals occurs when intraluminal or bacterial gas enters the portomesenteric circulation [11-13]. Necrotic bowel wall from infection, inflammation or ischemia and/or markedly increased intraluminal pressures seem to favor the entry of air into the venous radicals. In a large series of 64 patients with this finding, the reported mortality was 75%, nearly all patients requiring surgery [11]. Recent reviews have suggested that the mere finding of portal venous air by itself does not require surgery; it is important to treat patients based on their clinical condition [14].

## Conclusions

A case is presented in which emphysematous gastritis with portal venous air complicates cesarean section. Although this condition often requires surgery, this case resolved with appropriate medical management. To the best of our knowledge, this is the second report of emphysematous gastritis associated with portal venous air that was successfully treated without surgical intervention [15]. To date, there has been no reported association of emphysematous gastritis with neurofibromatosis.

## Consent

Written informed consent was obtained from the patient for publication of this case report and any accompanying images. A copy of the written consent is available for review by the Editor-in-Chief of this journal.

## Competing interests

The authors declare that they have no competing interests.

## Authors' contributions

SJ and MP evaluated the patient, reviewed the literature and drafted the article. MCM drafted the article, reviewed the literature and revised it critically. NHG and SLW interpreted the imaging studies, reviewed the literature and drafted the article pertinent to their filed of expertise. UKM supervised patient care, revised the articled critically and provided valuable inputs with regard to the management of the patient and final version of the draft. All authors read and approved the final manuscript.
